# Helminthic Infection in the Background of Active Pulmonary Tuberculosis: An Underreported Co-infection

**DOI:** 10.7759/cureus.13741

**Published:** 2021-03-06

**Authors:** Arshi Syal, Yajur Arya, Nikita N Hapani, Monica Gupta, Saurabh Gaba

**Affiliations:** 1 Internal Medicine, Government Medical College and Hospital, Chandigarh, IND

**Keywords:** tuberculosis, ascariasis, mebendazole, helminths, pulmonary

## Abstract

Helminthic infections are widely prevalent in resource-poor countries. Tuberculosis, a disease contributing significantly to morbidity and mortality in endemic areas, often co-exists with helminthic infections. Poor living standards predispose to both of them. Moreover, untreated helminthic infection enhances the deleterious impact of tuberculosis, largely through immunological alteration. We are reporting the case of a 22-year-old male who presented with a month-long history of abdominal pain, nausea, vomiting, fever and cough complicated by hemoptysis, along with an episode of the passage of a worm in the vomitus. A thorough workup revealed active pulmonary tuberculosis co-existing with intestinal ascariasis. Anti-helminthic therapy was initiated along with anti-tubercular therapy, leading to significant improvement.

## Introduction

Helminthic infections are widespread in resource-poor countries. Their impact is further augmented with concurrent tuberculosis in the background. The co-existence of helminthic infections and tuberculosis is significant but is often underreported [1]. An overlapping initial complex of constitutional symptoms can lead to a delay in the diagnosis of a co-existent helminthic infection. Various risk factors increase the propensity as well as susceptibility for contracting these infections. Overcrowding and unhygienic living conditions predispose individuals to tuberculosis as well as helminthic infections. Factors including living in a rural area, consumption of vegetables and fruits without washing or peeling and having a body mass index (BMI) of less than 18.5 kg/m^2^ have been significantly associated with an increased predisposition to intestinal helminthic infections [2]. A robust T-helper type 1 (Th1) response is required for resisting active tuberculosis infection. Helminths, on the other hand, induce a strong opposing T-helper type 2 (Th2) response along with total suppression of the immune system and preferential shift from the cell-mediated immune response toward humoral response [1]. These individuals are then predisposed to the development of active tuberculosis and suffer from increased morbidity. On the contrary, tuberculosis favors the immune escape of helminths, thereby propagating this vicious cycle [2].

## Case presentation

A 22-year-old male presented to the emergency room with hemoptysis. It was approximately 30 mL in volume and bright red in color. The episode occurred one hour prior to the presentation. It was not associated with any hypotension, dizziness, shortness of breath, history of prolonged immobility or recent surgery.

Two days prior to presentation, he had an episode of vomiting with the presence of a worm in vomitus. He also reported cough and fever for the preceding one month and a self-limiting episode of hemoptysis, approximately 15 mL in volume. The patient also complained of nausea and a vague, poorly localized central abdominal pain. Cough occurred in bouts and it was productive with straw-colored and mucoid sputum. There was history of intermittent fever, documented to be about 100-101 ºF, and evening rise of temperature associated with chills. There was no history of convulsions, the presence of any skin nodules or rash, any chronic ailment, contact with a patient of tuberculosis or previous anti-tubercular therapy (ATT) intake.

**Figure 1:**
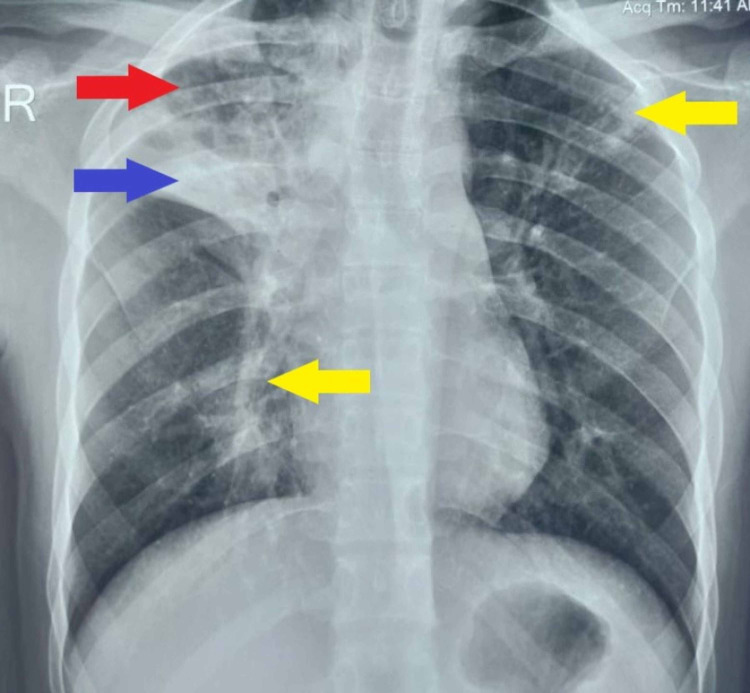
Radiograph of the chest showing a cavity (red arrow) and opacity (blue arrow) in the right upper zone along with bilateral areas of haziness (yellow arrows).

On initial evaluation, the patient was afebrile with a regular pulse rate of 95 per minute, blood pressure of 120/82 mm of Hg, respiratory rate of 14 per minute and oxygen saturation of 96%. His random blood sugar was 106 mg/dL. A radiograph of the chest (Figure 1) revealed right upper zone opacity with a cavity along with bilateral areas of haziness. The blood counts, coagulogram, electrolytes, liver and renal function tests were unremarkable.

**Figure 2:**
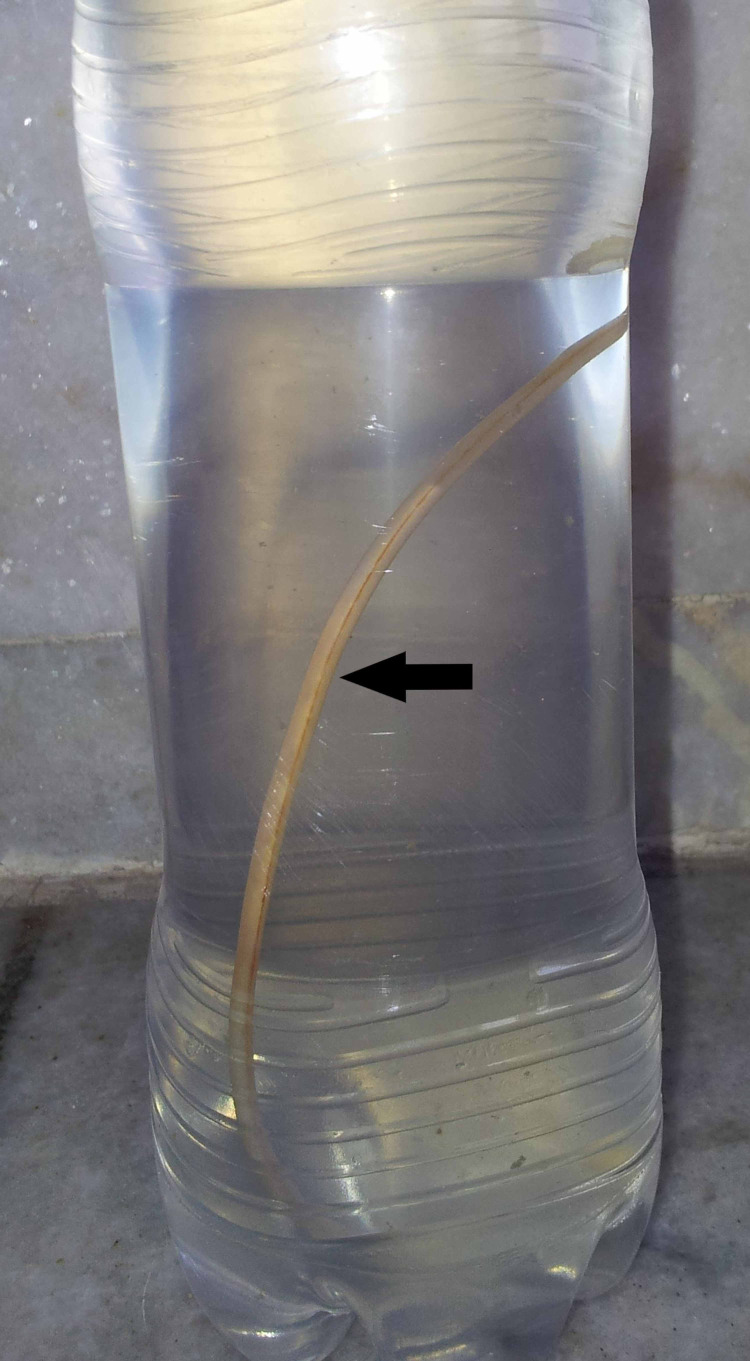
The worm (black arrow) that was present in the vomitus. Gross examination revealed it to be *Ascaris lumbricoides. *It was approximately 25 cm in length, light pink in color and tapering at both ends.

**Figure 3:**
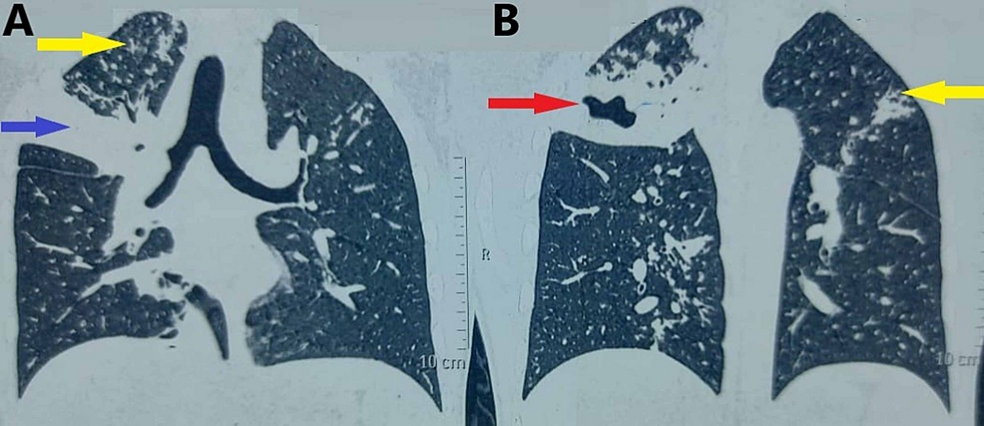
Coronal sections (A and B) of HRCT scan of the chest showing consolidation (blue arrow) and cavity (red arrow) in the right upper lobe along with bilateral branching centrilobular nodules (yellow arrows). HRCT: High-resolution computed tomography

**Figure 4:**
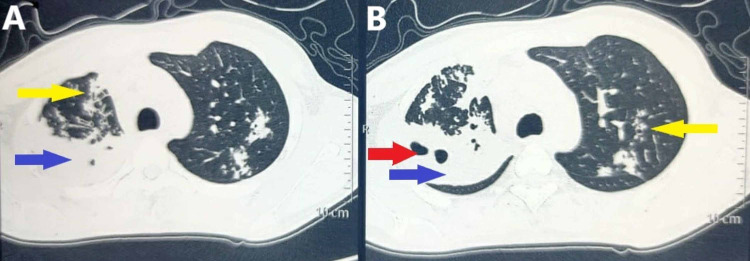
Axial sections (A and B) of HRCT scan of the chest showing consolidation (blue arrows) and cavity (red arrow) in the right upper lobe along with bilateral branching centrilobular nodules (yellow arrows). HRCT: High-resolution computed tomography

A diagnosis of ascariasis was made based on the examination of the worm (Figure 2). The patient was administered 100 mg mebendazole orally, twice daily for three days. Microbiological investigations of sputum, including gram stain, potassium hydroxide (KOH) stain for fungi and Ziehl-Neelsen (ZN) stain for acid-fast bacilli were negative on three occasions, and culture failed to detect any organism. A high-resolution computed tomography (HRCT) scan of the chest (Figures 3A, 3B, 4A, 4B) revealed a large patch of consolidation with central cavitation in the upper lobe of the right lung and bilateral multiple centrilobular nodules with linear branching (tree-in-bud sign). These changes were suggestive of an active tubercular infection.

A sputum cartridge-based nucleic acid amplification test (CBNAAT) was performed, which was positive for *Mycobacterium tuberculosis* and a diagnosis of active pulmonary tuberculosis was thus confirmed. Further management included initiation of a weight-based ATT regimen, comprising isoniazid, rifampicin, ethambutol and pyrazinamide along with pyridoxine supplementation. Routine monitoring of parameters including complete blood count, liver and renal function tests was performed. Symptomatic improvement started during the second week of ATT intake. He was discharged in a stable condition with continuation of ATT for a total duration of six months.

## Discussion

The enhanced production of Th2 and T-regulatory cells along with consequential cytokine surge involving interleukins 4 and 10 with transforming growth factor-beta (TGF-beta) generated due to helminthic infection may be responsible for inhibition of Th1 response against *M. tuberculosis*. Co-infection with helminths can predispose toward the development of active *M. tuberculosis* infection, enhanced dissemination and development of sequelae [3]. The risk of development of active tuberculosis can be increased by as much as two-fold [4]. Also, increased generation of interleukin 10 and TGF-beta has been associated with the reactivation of latent tuberculosis [5]. Th2 upregulation is responsible for the enhanced production of mucus and collagen in an attempt to eliminate helminths. However, these mechanisms are not sufficient, often resulting in chronic infection [6]. Rather, activation of these mechanisms can surprisingly lead to the persistence of helminths consequential to immunological suppression by enhanced production of T-regulatory cells, a process that is further exaggerated by Th2 cells. Furthermore, Th2 cells lead to anergy of immunological cells, thereby compromising crucial cell-mediated immune response against *M. tuberculosis* [7].

Chronic infections can lead to anemia, growth retardation and impaired cognitive development. A BMI of less than 18 kg/m^2^, involvement in raising poultry or livestock, having anemia and walking barefoot in farmlands are associated with intestinal helminthic infections [8]. Exposure to helminths during pregnancy is postulated to prime the neonate toward greater production of Th2 cells. This might be a factor leading to a lower Bacillus Calmette-Guerin (BCG) immunogenicity later in life [9]. Also, these infections might compromise the sensitivity of tuberculosis diagnostic tests by reducing interferon-gamma (IFN-gamma) levels [10].

Diagnosis of ascariasis is usually established by demonstration of eggs in feces or the adult worm in stool, vomitus or sputum. Anti-helminthic therapy forms the mainstay of treatment. Not only does this regimen eradicate helminths, but also reverses the altered IFN-gamma levels, thereby improving diagnostic sensitivity [11]. Benzimidazole class of antiparasitic agents is excellent therapeutic modalities. These drugs inhibit the polymerization of tubulin and microtubule-dependent glucose uptake in these parasites [12]. Albendazole (single oral dose of 400 mg) or mebendazole (single oral dose of 500 mg or 100 mg twice daily for three days) form the cornerstone of management in adult non-pregnant individuals, although it might not prevent reinfection [13]. Adverse effects like nausea, vomiting, abdominal pain and elevation of transaminases might occur, but it does not warrant cessation of therapy. Concurrent ATT administration for tuberculosis does not need to be withheld. Follow-up testing is usually not required but can be done by stool egg counts, approximately 14 days after therapy [14]. Strategies for control include improvements in sanitation, health education and mass anti-helminthic treatment [15]. Thus, taking a healthy and balanced diet, prevention of nutritional deficiencies and imparting education and creating awareness regarding personal hygiene are necessary to decrease the burden of helminthic infections in patients with tuberculosis.

## Conclusions

Our case highlights the need to be cognizant of the possibility of co-existent helminthic and tubercular infections, especially in the high prevalence and resource-limited regions. An overlap in the clinical presentation of both of these conditions in the form of anorexia, fever, malaise and abdominal pain might lead to a delay in diagnosis. This delay should be avoided by suitable investigations in order to prevent an adverse clinical outcome. Anti-helminthic therapy forms the mainstay of management of ascariasis and ATT can be initiated concurrently.
